# Ferroptosis targeting natural compounds as a promising approach for developing potent liver cancer agents

**DOI:** 10.3389/fphar.2024.1399677

**Published:** 2024-04-26

**Authors:** Pratibha Pandey, Deena Elsori, Rahul Kumar, Sorabh Lakhanpal, Indra Rautela, Tariq Mohammed Alqahtani, Fuzail Ahmad, Danish Iqbal, Fahad Khan

**Affiliations:** ^1^ Centre of Research Impact and Outcome, Chitkara University, Rajpura, Punjab, India; ^2^ Faculty of Resilience, Rabdan Academy, Abu Dhabi, United Arab Emirates; ^3^ Chitkara Centre for Research and Development, Chitkara University, Baddi, Himachal Pradesh, India; ^4^ School of Pharmaceutical Sciences, Lovely Professional University, Phagwara, Punjab, India; ^5^ School of Applied and Life Sciences, Uttaranchal University, Dehradun, India; ^6^ Department of Radiology and Medical Imaging, College of Applied Medical Sciences, Majmaah university, Al Majma’ah, Saudi Arabia; ^7^ Respiratory Care Department, College of Applied Sciences, Almaarefa University, Riyadh, Saudi Arabia; ^8^ Department of Health Information Management, College of Applied Medical Sciences, Buraydah Private Colleges, Buraydah, Saudi Arabia; ^9^ Center for Global Health Research, Saveetha Medical College and Hospital, Saveetha Institute of Medical and Technical Sciences, Chennai, Tamil Nadu, India

**Keywords:** ferroptosis, liver cancer, therapeutics, natural product, nanomaterials, hepatocellular carcinoma

## Abstract

Liver cancer is the second leading cause of cancer-related death worldwide. However, treatment options, including surgical resection, transplantation, and molecular drug therapies, are of limited effectiveness. Recent studies have demonstrated that suppressing ferroptosis might be a pivotal signal for liver cancer initiation, thus providing a new way to combat liver cancer. Ferroptosis is a distinct form of controlled cell death that differs from conventional cell death routes like apoptosis, necrosis, and pyroptosis. It results from intracellular iron overload, which raises iron-dependent reactive oxygen species. This, in turn, leads to the accumulation of lipid peroxides that further result in oxidative damage to cell membranes, disrupt normal functioning, and ultimately speed up the ferroptosis phenomenon. Ferroptosis regulation is intricately linked to cellular physiological processes, encompassing iron metabolism, lipid metabolism, and the equilibrium between oxygen-free radical reactions and lipid peroxidation. This review intends to summarize the natural compounds targeting ferroptosis in liver cancer to offer new therapeutic ideas for liver cancer. Furthermore, it serves as the foundation for identifying and applying chemical medicines and natural chemicals that target ferroptosis to treat liver cancer efficiently.

## 1 Introduction

Liver cancer and ferroptosis are closely associated with metabolic abnormalities. Metabolic disorders are directly associated with liver cancer (Zhang et al., 2019a). Liver cancer is characterized by abnormal activation of glucose metabolism, including glycolysis and hexosamine production, which enhance malignant characteristics. Lipid peroxide-induced cell death is known as ferroptosis and is caused by the suppression of glutathione (GSH) synthesis (Chen et al., 2022a). In 2012, ferroptosis was originally recognized as a new type of RCD, or regulated cell death (Dixon et al., 2012). The condition is distinguished by an overabundance of intracellular lipid reactive oxygen species (ROS) and lipid peroxidation, which arises from the depletion of glutathione in response to iron and the inactivation of glutathione peroxidase 4 (GPX4) (Mou et al., 2019). This novel form of cell death is morphologically, genetically, and biochemically different from existing recognized types of cell death such as apoptosis, necroptosis, pyroptosis, and autophagy. The key processes of ferroptosis include elevated iron levels, dysfunctional lipid repair mechanisms, and lipid peroxidation, resulting in membrane damage and cell demise (Gao et al., 2016). The three most often reported indicators of ferroptosis are lipid peroxidation, elevated prostaglandin synthase 2 (PTGS2) expression, and reduced levels of the active form of nicotinamide adenine dinucleotide phosphate (NADPH), despite the absence of recognized criteria for its occurrence (Lei et al., 2019).

The progression of liver cancer is strongly associated with the inhibition of ferroptosis (Wu et al., 2021). Liver cancer cells can be destroyed through ferroptosis, and erastin has been identified as capable of triggering ferroptosis and causing liver cancer cell death (Yu et al., 2017). An enhanced comprehension of the mechanism controlling ferroptosis could aid in the development of therapeutic drugs for liver cancer. Reactive oxygen species are oxygen-containing compounds that exhibit chemical reactivity and decrease liver cancer. Exposure to erastin and other external substances can cause an increase in reactive oxygen species, resulting in a kind of cell death called ferroptosis (Čepelak et al., 2020). Ferroptosis can be induced by inhibiting glutathione synthesis or by overwhelming the system with iron. In liver cancer, the nuclear factor erythroid 2-related factor 2 (NRF2) transcription factor (TF) stimulates the expression of several reductases that can inhibit ferroptosis by reducing ROS accumulation, and an increase in the proto-oncoprotein p62 inhibits NRF2 degradation, which in turn boosts NRF2 transcription activity (Chen et al., 2023; Yan et al., 2023). Metallothionein (MT)-1G has been found to decrease GSH depletion and lipid peroxidation, hence preventing ferroptosis in liver cancer (Houessinon et al., 2016). Irrespective of the subtypes, liver cancer has a grim prognosis because it is often not detected at treatable stages and lacks effective curative treatments.

Hepatic resection and liver transplantation are the primary treatments for liver cancer; however, only a small number of patients are eligible. Treatment options for most patients in the advanced stages typically involve ablation therapies, transarterial chemoembolization (TACE), radiation therapy, and systemic medications (Vogl et al., 2009; Hernaez and El-Serag, 2018; Li and Ni, 2019; Miyayama, 2020). Multikinase inhibitors, such as sorafenib and regorafenib, have been authorized as the primary treatment for patients with advanced hepatocellular carcinoma (HCC) (Raoul et al., 2018). Nevertheless, these medications only extend the median lifespan of patients with advanced HCC by approximately 3 months. Low response and high recurrence rates are significant challenges in the treatment of liver cancer (Anwanwan et al., 2020). Further research is urgently required to develop more effective therapeutic approaches for liver cancer patients. Currently, most liver cancer patients are diagnosed in the middle to late stages, and the accuracy of liver cancer biomarkers is not particularly high. Most studies have shown that using a panel of biomarkers together with traditional alpha-fetoprotein (AFP) significantly improves diagnostic precision and sensitivity (Hanif et al., 2022). As inhibiting ferroptosis is strongly linked to the development of liver tumors, blood biomarkers indicating ferroptosis suppression could be useful for diagnosis. We are not explaining ferroptosis in detail as our recently published article has outlined a detailed mechanism for the involvement of ferroptosis in cancer progression (Khan et al., 2024). Natural compounds are gaining enormous attention in cancer therapeutics because they target numerous deregulated tumor oncogenes to develop potential drug candidates with limited toxicity and side effects. Therefore, our review has highlighted ferroptosis in liver cancer and enlisted all possible natural compounds targeting ferroptosis in liver cancer which would further help future researchers to elucidate potent antihepatocellular carcinoma agents.

## 2 Ferroptosis in liver cancer

The liver is crucial for regulating iron balance in the body. The liver utilizes iron mobilization to maintain hepatic iron levels by regulating iron transportation and storage through gene control (Milic et al., 2016; Wang and Babitt, 2019). Iron is a crucial micronutrient for numerous fundamental physiological functions. Excessive iron can damage lipids, DNA, and proteins leading to cell rupture and death. It is essential to maintain the ratio of iron supply and iron utilization. The liver organ is where iron is stored in the body and is essential for controlling iron-related hormones to ensure proper iron levels (Daher et al., 2017). Liver dysfunction causes disturbances in iron equilibrium, leading to different iron-related disorders such as anemia and iron overload. Various phases of liver illness display ferroptosis traits such as accumulation of lipid peroxides, disruption in iron metabolism, and imbalance in the amino acid antioxidant system (Mao et al., 2020). To prevent the development of various liver illnesses, one can focus on addressing ferroptosis at a pathophysiological level. We have not elaborated on the ferroptosis mechanism in detail, as several articles have presented extensive reviews deciphering novel insights into ferroptosis modulation as a potential strategy for cancer treatment (Consoli et al., 2024). Hence, we have focused on compiling the latest research findings to enlist all reported natural compounds targeting ferroptosis in hepatocellular carcinoma to create new therapeutic approaches for liver carcinoma.

**FIGURE 1:**
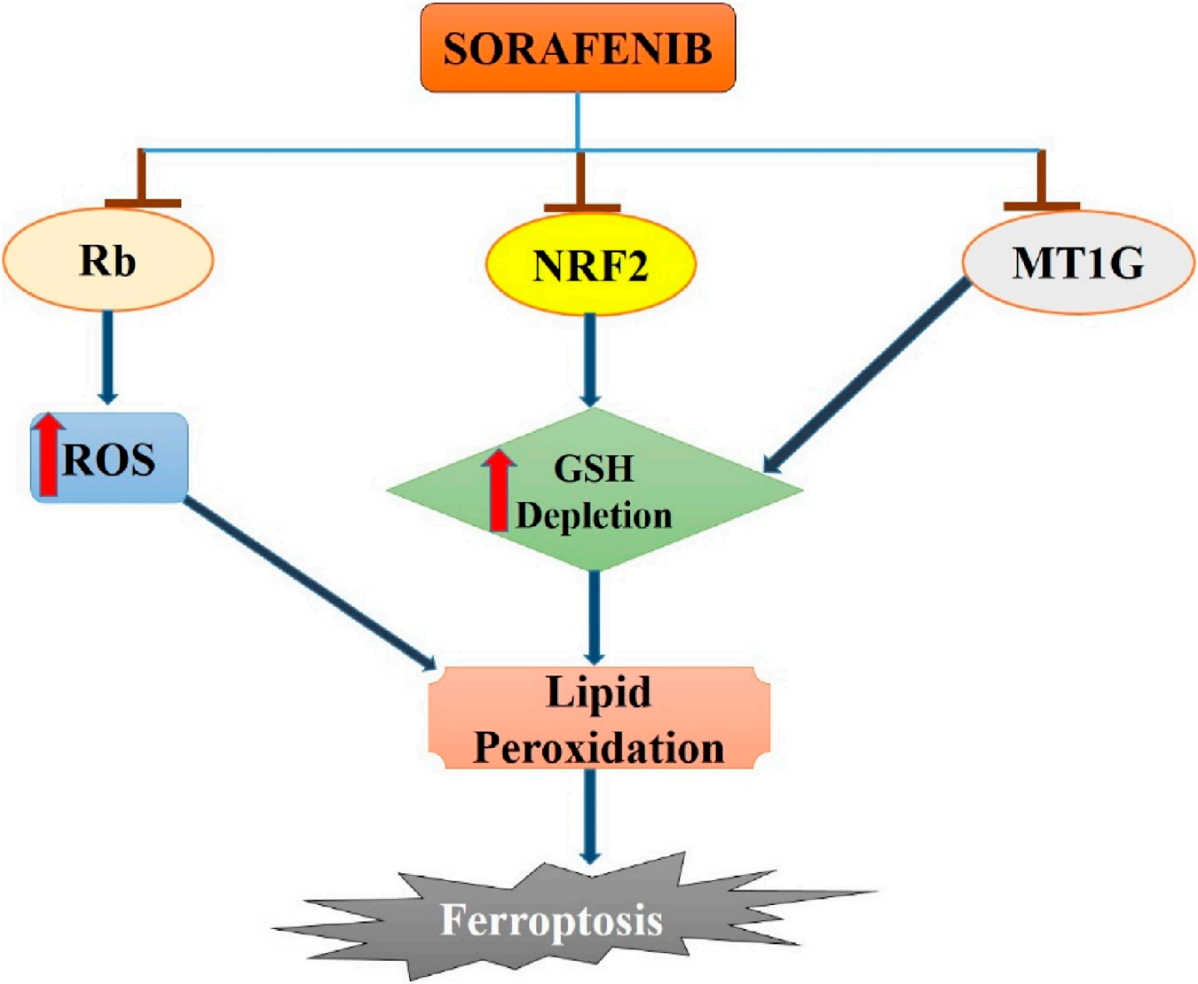
Sorafenib-induced ferroptosis in hepatocellular carcinoma. Sorafenib blocks the activity of Rb, NRF2 and MT1G in hepatocellular carcinoma cells which leads to increase in ROS generation and GSH depletion. Enhanced ROS and GSH depletion results in lipid peroxidation and then to ferroptosis induction in hepatocellular cancer.

One of the leading causes of cancer-related deaths globally is hepatic carcinoma, which has a poor prognosis because patients are frequently diagnosed in the advanced stages of the disease. Over 90% of reported liver cancer cases are hepatocellular carcinoma (Anwanwan et al., 2020). Suppression of ferroptosis is closely linked to the progression of liver cancer. Recent studies have demonstrated that ferroptosis activators can inhibit the antioxidant glutathione, either directly or indirectly through several pathways. This results in the excessive accumulation of lipid reactive oxygen species and, ultimately, cell death. Recent studies have demonstrated that blocking ferroptosis can play a significant role in the initiation of liver cancer, providing a new strategy to fight this illness (Liu et al., 2022a). Following phosphorylation, p62 binds to Keap1 and dissociates NRF2 from Keap1, leading to activation of the Keap1-NRF2-ARE pathway. This system is associated with antioxidant defense, survival, and metabolic reprogramming in hepatocellular carcinoma (Dodson et al., 2019). Erastin, a frequent inducer of ferroptosis, inhibits SLC7A11, a component of the cystine/glutamate antiporter. This inhibition causes a reduction in glutathione (GSH) synthesis, leading to the accumulation of reactive oxygen species and ferroptosis. GPX4 plays a vital role in the inhibition of ferroptosis in several cancer types. GPX4 is a selenoprotein with a vital selenocysteine residue in its catalytic domain (Lee and Roh, 2022). Ebselen, a compound that mimics the function of GPX4, provides partial protection against ferroptosis in HCC cells. Ferroptosis is a newly identified form of cellular death that has the potential to identify new therapeutic targets and predict the outcome of hepatocellular carcinoma (Chen et al., 2021). Sorafenib, a multi-kinase inhibitor, has long been the gold standard for the management of advanced HCC (Figure 1). In 2013, researchers demonstrated that sorafenib could trigger ferroptosis, leading to its classification as a ferroptosis inducer (Louandre et al., 2013).

Several studies have emphasized the important role of ferroptosis in HCC and the strong connection between certain ferroptosis modulators, such as p53, retinoblastoma (Rb) protein, and NRF2, and cancer development. Research has investigated the mechanism of acquired resistance to sorafenib in HCC patients to reverse or prevent it, while also exploring new insights into ferroptosis cell death (Tang et al., 2020). Recent evidence indicates that MT-1G plays a crucial role as a ferroptosis suppressor. Inhibiting MT-1G genetically or pharmacologically enhances the anticancer effects of sorafenib in both laboratory settings and tumor models. This inhibition leads to reduced GSH levels and increased lipid peroxidation without affecting cellular iron content (Guo et al., 2023).

It is noteworthy that ferroptosis appears to be mediated by the sigma-1 receptor (r1R), as evidenced by the observation that the r1R antagonist haloperidol stimulates erastin-induced ferroptotic cell death in HCC cells (Bai et al., 2017). According to a study by Zhang et al. (2019b), stimulation of ferroptosis results in a change in the ratio of HIC1 to HNF4A, which may be helpful as a novel approach for the treatment of liver cancer and may prevent the formation of cancer. As previously reported for CRC, a study by Liang et al. (2020) established a prognostic model composed of 10 ferroptosis-related genes also for HCC: GPX4, NAD(P)H Quinone Dehydrogenase 1 (NQO1), Solute Carrier Family 1 Member 5 (SLC1A5), Acetyl-CoA Carboxylase Alpha (ACACA), Acyl-CoA Synthetase Lon Chain Family Member 3 (ACSL3), CISD1, Cysteinyl-TRNA Synthetase 1 (CARS1), Glucose-6-Phosphate Dehydrogenase (G6PD), GPX4, and Solute Carrier Family 1 Member 5 (SLC1A5).

RRM2 (ribonucleotide Reductase Regulatory TP53 Inducible Subunit M2B) has been identified as a tumor biomarker associated with ferroptosis in liver cancer (Yang et al., 2020a), and Tang et al. (2021) used sustained GSH production to show that RRM2 has an anti-ferroptotic effect. Additionally, the association between elevated RRM2 serum levels and advanced tumor stage in liver cancer suggests that it could serve as a promising indicator of sensitivity to ferroptosis and as a factor that can be targeted for cancer therapy. He et al. (2021) discovered that ketamine could trigger ferroptosis in liver cancer by controlling the lncPVT1/miR-214-3p/GPX4 pathway. It has been shown that the overexpression of LncRNA plasmacytoma variant translocation 1 (PVT1), which is associated with multiple cancer types, can modulate the expression levels of GPX4 by suppressing miR-214-3p, hence promoting ferroptosis inhibition in liver cancer. Ketamine therapy had a detrimental effect on lncPVT1 levels, which caused liver cancer cells to undergo ferroptosis and demonstrated the potential advantages of using the lncPVT1/miR-214-3p/GPX4 axis as a target for groundbreaking therapeutics (He et al., 2021).

## 3 Natural compounds targeting ferroptosis in liver cancer

Nature serves as an abundant source of novel chemicals that may possess significant biological activity. Currently, the scientific community is showing significant interest in the exploration of natural sources for the identification of novel medicinal molecules (Cragg and Newman, 2013; Thomford et al., 2018). Furthermore, the advancement of contemporary methodologies for the discovery of natural products as drugs could accelerate the process of identifying new compounds with the potential to be utilized in pharmacological treatments (Cichonska et al., 2015). The utilization of phytochemical compounds offers significant benefits owing to their availability, possibility of reduced toxicity, and diverse range of structural variations. This diversity enables the identification of common frameworks responsible for specific therapeutic effects (Singh et al., 2019; Pham et al., 2020). In this article, we present an overview of the most effective natural compounds that promote ferroptosis in liver cells along with their mechanisms of action. These compounds show great potential for the development of novel anticancer treatments. Table 1 summarizes all reported natural compounds targeting ferroptosis in liver cancer.

**TABLE 1:** List of natural compounds inducing ferroptosis in hepatocellular carcinoma.

Class of the natural compound	Name of the natural compound	Mode of action	References
Pentacyclic triterpene acid	Corosolic acid	Increased sensitivity to ferroptosis via upregulating HERPUD1	Peng et al. (2022)
Alkaloids	Solasonine	Ferroptosis induction	Jin et al. (2020)
		Enhanced ROS	
Alkaloids	Sophoridine derivative 6j	ATF3-mediated ferroptosis	Tian et al. (2023)
		Enhanced accumulation of intracellular Fe2+	
		Increased reactive oxygen species level and MDA levels	
Benzophenanthridine alkaloid	Sanguinarine	Reduced stability of GPX4 protein	Xu et al. (2022)
		Ferroptosis induction	
Natural flavonoid	Apigenin	Enhanced autophagy	Liang et al. (2023a)
		The hindered proliferation of tumor tissues	
Natural flavonoid	Quercetin	Enhanced ferritin breakdown	Wang et al. (2021)
		Bid-mediated ferroptosis	
		Induced ROS production	
Natural flavonoid glycoside	Silibinin	Ferroptosis induction	Song et al. (2022)
		Alleviated oxidative stress	
		Reduced iron levels	
Natural flavonoid glycoside	Tiliroside	Enhanced efficacy of sorafenib	Yang et al. (2023)
		Keap1-mediated NRF2 ubiquitination	
		Hindered development of HepG2 tumors	
		Triggered Ferroptosis	
Flavonoid	Scutellaria barbata	Inhibition of cell proliferation	Li et al. (2022a)
		Ferroptosis induction	
		Increased iron peroxidation	
		Enhanced lipid ROS metabolism	
Terpenoid	Caryophyllene oxide	Enhanced ROS generation	Jin et al. (2023)
		Increased lipid peroxidation	
		Enhanced ferritinophagy	
		Reduced tumor volume	
Marine terpenoid	Heteronemin	Triggered cell death through the caspase pathway	Chang et al. (2021)
		Enhanced ROS generation	
		Ferroptosis induction	
Sesquiterpene	Parthenolide	Ferroptosis induction	Kreuger et al. (2012)
		Increased ROS generation	
		Increased mitochondrial dysfunction	
		Increased lipid peroxidation	
	Gallic acid	Inducing ferroptosis	Xie et al. (2023)
		Deactivation of the Wnt/β-catenin signaling pathway	
Saponin	Formosanin C	Cell viability inhibition	Lin et al. (2020); Su and Lin (2020)
		Enhanced lipid ROS production	
		Triggered autophagic flux	
		Enhanced ferritinophagy	
Steroidal saponin	SSPHI	Antiproliferative and anti-migratory effects	Huang et al. (2023)
		Triggered ferroptosis	
		Cell cycle arrest at the G2/M phase	
		Enhanced ROS generation	
		Glutathione depletion	
		Malondialdehyde accumulation	
Triterpenoid saponin	Saikosaponin A	Elevated intracellular malondialdehyde and iron levels	Lan et al. (2023)
		Reduced glutathione levels	
		Ferroptosis induction	
		ATF3-dependent cell ferroptosis	

Corosolic acid (CA), a monomer derived from the root plant Actinidia valvata Dunn, exhibits numerous beneficial properties, including anti-diabetic, weight-loss-enhancing, anti-inflammatory, antiviral, anticancer, and anti-cardiovascular effects. CA increases the sensitivity of liver cancer cells to ferroptosis, a controlled type of cell death characterized by the accumulation of iron-dependent lipid peroxides to deadly levels. CA hinders the production of GSH through the action of HERPUD1, resulting in a reduction in cellular GSH levels and an increase in the susceptibility of liver cancer cells to ferroptosis. HERPUD1 decreased the process of attaching ubiquitin molecules to the GSS-associated E3 ubiquitin ligase MDM2, which enhanced the process of attaching ubiquitin molecules to GSS. This inhibits GSH synthesis, leading to increased vulnerability to ferroptosis. CA has been shown to decrease tumor growth in a mouse xenograft model by targeting HERPUD1. This suggests that CA is a potential candidate for the development of liver cancer therapy (Peng et al., 2022).

### 3.1 Alkaloids as ferroptosis inducer in liver cancer

Alkaloids are a broad class of molecules with a nitrogen atom that have a variety of biological effects, such as analgesic, anesthetic, antimalarial, antiarrhythmic, and anticancer properties (Mondal et al., 2019; Peng et al., 2022). Compounds from this chemical class have been reported to exhibit proferroptotic characteristics in certain cancer cell lines. In a xenograft model of HCC, solasonine therapy caused ferroptosis by interfering with the function of the GSH redox system, thereby increasing ROS levels (Jin et al., 2020; Kusnick et al., 2022). The sophoridine derivative 6j (selective inhibitor of discoidin domain receptor 1) hinders the proliferation of liver cancer cells (HepG2 and MHCC-97H cell lines) through ATF3-mediated ferroptosis. ATF3, which belongs to the ATF/CREB family of transcription factors, is extensively present in normal human tissues, such as the liver, gastrointestinal system, and endocrine tissues (Chen et al., 2022b). Administration of ferrostatin-1 resulted in reduced accumulation of Fe^2+^, MDA, and ROS induced by compound 6j, thereby promoting the restoration of cell viability through the inhibition of ferroptosis. Furthermore, there was a significant reduction in tumor weight in 6j treatment groups (High and low dose) compared to the vehicle treatment group. Following the administration of 6j in a dose-dependent manner, a significant increase in ATF3 expression was observed in a xenograft mice model (Tian et al., 2023).

Sanguinarine (SAG) is a benzophenanthridine alkaloid found in the root of Sanguinaria canadensis Linn. (Bloodroot), which shows promise as an anti-cancer agent (Zhang et al., 2019c). SAG reduced the protein stability of GPX4 by promoting ubiquitination and breakdown of endogenous GPX4 through the E3 ligase STUB1. GPX4 overexpression reversed the inhibition of proliferation and invasion of NSCLC cells treated with SAG via suppression of ferroptosis. SAG hinders the development and spread of non-small cell lung cancer by controlling STUB1/GPX4-dependent ferroptosis (Xu et al., 2022).

### 3.2 Flavonoids as ferroptosis inducer in liver cancer

Flavonoids are a diverse group of phytocompounds that contain a 15-carbon backbone within a 2-phenylbenzopyranone scaffold. Flavonoids can further be classified into various subclasses, including flavones, flavonols, flavanols, isoflavones, anthocyanidins, flavanones, and chalcones, based on the level of unsaturation of the pyranone ring and the presence of oxygen-containing functional groups (Shen et al., 2022). In addition, sugars attached to the heterocyclic backbone enable further classification into free aglycones and glycoside derivatives. Research has focused on identifying flavonoids that can influence the ferroptotic process, as they are generally harmless (Zhang et al., 2021). Apigenin, (natural flavonoid) is highly effective against numerous human carcinomas. There is limited research showing the efficacy of apigenin in ferroptosis and autophagy induction in Ishikawa endometrial carcinoma cells (Liang et al., 2023a). Iron build-up, glutathione consumption, lipid peroxidation, ferritin, p62, and HMOX1 levels were elevated, whereas solute carrier family seven member 11 and glutathione peroxidase four levels were reduced. Ferrostatin-1 (iron-death inhibitor), counteracts the effects of apigenin in Ishikawa cells. Apigenin can enhance autophagy by increasing the expression of Beclin 1, ATG13, ULK1, LC3B, and ATG5 while decreasing the levels of AMPK, P70S6K, ATG4, and mTOR. Apigenin can hinder the proliferation of tumor tissues and limit tumor growth through ferroptosis in mice models (Zhang et al., 2023a; Bi et al., 2023).

Quercetin has been reported to induce cell death in several cancer cells without relying on the p53 protein. Quercetin triggers lysosome activation by EB and enhances ferritin breakdown, resulting in Bid-mediated ferroptosis and apoptosis. Quercetin notably enhances the breakdown of ferritin in lysosomes, leading to the release of free iron. The combination of this activity and quercetin-induced ROS production leads to lipid peroxidation and ferroptosis in a synergistic manner. Moreover, Bid may be associated with ferroptosis and apoptosis to induce cell death (Wang et al., 2021). Silibinin is a traditional liver protectant derived from the natural flavonoid glycoside. This study investigated how silibinin modulates ferroptosis to protect liver cells from harm generated by ethanol or acetaldehyde, utilizing human HepG2 liver cancer cells and HL7702 immortalized liver cells. Silibinin rescues ferroptosis triggered by ethanol or acetaldehyde through NCOA4-dependent autophagic destruction of ferritin, a protein responsible for iron storage. Increased degradation of ferritin and elevated levels of reactive oxygen species occurs when PINK1 or Parkin are suppressed in cells exposed to ethanol or acetaldehyde (Song et al., 2022).

Another study investigated whether tiliroside, a natural flavonoid glycoside extracted from oriental paperbush flowers, enhanced the responsiveness of hepatocellular carcinoma cells to sorafenib. Tiliroside greatly enhances the efficacy of sorafenib against hepatocellular carcinoma with minimal side effects (Yang et al., 2023). Additionally, the combination of tiliroside and sorafenib has demonstrated synergistic benefits against hepatocellular carcinoma. Tiliroside has been shown to directly bind to TANK-binding kinase 1 (TBK1) and blocks its enzymatic activity which further promotes Keap1-mediated NRF2 ubiquitination and destruction. The downstream target proteins of NRF2, such as glutathione peroxidase 4, ferritin heavy chain 1, and glucose-6-phosphate dehydrogenase, showed comparable outcomes to NRF2 protein, leading to ferroptosis in tiliroside-treated HCC cells. Tiliroside hindered the development of HepG2 tumors in both subcutaneous and orthotopic xenograft tumor models of HCC which further enhanced the effectiveness of sorafenib in treating hepatocellular carcinoma by targeting TBK1 to trigger ferroptosis (Yang et al., 2023). Ferroptosis is initiated by the accumulation of iron-dependent lipid peroxidation, which results in a reduction in cell volume and an increase in mitochondrial membrane density. Ferroptosis is involved in the pathogenesis of important illnesses such as hepatocellular carcinoma. Traditional Chinese Medicine (TCM) is a unique medical resource widely used to treat HCC. This study revealed that S. barbata triggers ferroptosis in HCC cells by increasing iron peroxidation and lipid ROS metabolism in HCC cells (Li et al., 2022a).

### 3.3 Terpenoids as ferroptosis inducer in liver cancer

Another study found that caryophyllene oxide significantly inhibited HCCLM3 and HUH7 cells by modulating cellular oxidative stress, autophagy, and iron metabolism levels, resulting in a ferritinophagy-related impact. Caryophyllene oxide was found to enhance the generation and buildup of intracellular reactive oxygen species and lipid peroxidation during the evaluation of ferritinophagy-related process (Jin et al., 2023). Caryophyllene oxide controlled the expression of NCOA4, LC3 II, and FTH1 to enhance ferritinophagy. Caryophyllene oxide treatment reduced tumor volume enhanced NCOA4 and LC3 protein levels in tumor tissues, and increased Fe^2+^ and malondialdehyde levels in the blood. Caryophyllene oxide reduced the expression levels of NRF2, GPX4, HO-1, and FTH1, which led to decreased GSH and hydroxyl radical inhibitory capabilities in serum. This also causes iron accumulation in the tumor tissue, leading to the prevention of tumor growth (Xiu et al., 2022). Caryophyllene oxide primarily eliminated liver cancer cells by inducing ferroptosis through ferritinophagy. Caryophyllene oxide can serve as an activator of ferritinophagy in anticancer medication research and development.

Heteronemin, a marine terpenoid extracted from Hippospongia sp., has demonstrated protective effects against carcinogenesis in cholangiocarcinoma, prostate cancer, and acute myeloid leukemia (Yang et al., 2020b). Heteronemin was discovered to hinder the growth of HCC cell lines HA22T and HA59T and trigger cell death through the caspase pathway. The MAPK signaling pathway is linked to cell death triggered by ROS, and heteronemin reduces the production of ERK, a MAPK associated with cell growth. Heteronemin therapy decreases the expression of GPX4, a protein that suppresses ferroptosis, a unique type of non-apoptotic programmed cell death. Heteronemin is a potent drug that induces cell death in hepatocellular carcinoma cells through the activation of intracellular reactive oxygen species and the p38/JNK MAPK signaling pathway (Chang et al., 2021). This revealed a strong connection between apoptosis and ferroptosis mediated by MAPK signaling.

Parthenolide (PTL) is a sesquiterpene lactone compound naturally found in feverfew plants (Tanacetum parthenium). Parthenolide triggers rapid thiol oxidation, resulting in ferroptosis in hepatocellular cancer cells. Parthenolide (PTL) is a naturally occurring medicinal compound with anticancer and anti-inflammatory activities (Kreuger et al., 2012). PTL can alter HCC’s antioxidant environment of HCC by modifying thiols, making tumor cells more susceptible to increased reactive oxygen species. Studying the relationship between changes in the thiol pathway and heightened susceptibility to iron-induced lipid peroxidation can lead to enhanced HCC therapy. HepG2 (human) and McARH7777 (rat) HCC cells exposed to PTL at increasing doses exhibited reduced cell viability and clonogenic efficiency *in vitro*. PTL enhances the oxidation of glutathione, which can be reversed by adding the GSH precursor N-acetylcysteine (NAC) (LoBianco et al., 2022). Furthermore, this increase in thiol oxidation leads to an increase in mitochondrial dysfunction. Investigating the mechanism of cell death involves assessing lipid peroxidation. The results from a lipid peroxidation sensor showed that PTL elevated lipid oxidation levels after 6 h. Western blotting showed a decrease in GPX4 protein levels following treatment with PTL, indicating that cells are unable to prevent lipid peroxidation after exposure to PTL. Increased lipid peroxidation results in cell death, known as ferroptosis. The introduction of ferrostatin-1 along with PTL partially restored cell survival in the colony survival test. This study showed that PTL can cause tumor cell death by increasing intracellular oxidation levels, making cells susceptible to ferroptosis (LoBianco et al., 2022).

### 3.4 Saponins as ferroptosis inducer in liver cancer

Gallic acid, a natural phenolic phytocompound, hindered the growth and advancement of certain human cancers (Bonta, 2020). Inducing ferroptosis in tumor cells is currently recognized as one of the most efficient ways to eradicate them. Gallic acid treatment induced ferroptosis in HepG2 cells by inhibiting the synthesis of ferroptosis-related proteins SLC7A11 and GPX4. This hindered the movement of β-catenin from the nucleus to the cytoplasm, resulting in the deactivation of the Wnt/β-catenin signaling pathway. Gallic acid was identified as a novel trigger of ferroptosis in liver cancer (Xie et al., 2023). Saponins are common dietary components present in different edible plants such as soybeans and chickpeas. Formosanin C (FC) is a diosgenin saponin used in traditional Chinese medicine to treat snake venom toxicity and cysts. Formosanin C (FC) was discovered as a new ferroptosis inducer by screening for natural chemicals. It is distinguished by its capacity to reduce FC-induced viability inhibition and lipid ROS production in the presence of a ferroptosis inhibitor (Lin et al., 2020). FC triggered autophagic flux by inhibiting the degradation of the autophagic marker LC3-II and increasing the presence of yellow LC3 puncta in cells transfected with a tandem fluorescent-tagged LC3 (mRFP-GFP) reporter plasmid (ptfLC3) when used in combination with an autophagic flux inhibitor. FC-induced ferroptosis and autophagic flux were more pronounced in HepG2 cells with elevated NCOA4 and reduced ferritin heavy chain 1 (FTH1) levels (Su and Lin, 2020). This aligns with the gene expression analysis results from CTRP and PRISM, showing a significant negative correlation between FTH1 expression and cell sensitivity to ferroptosis inducers. Confocal and electron microscopy confirmed the significant role of ferritinophagy in ferroptosis induced by ferroptosis inducers in cells with increased NCOA4 levels. This study proposes that ferroptosis, a type of cell death independent of apoptosis, indicates that FC may have potential as a chemotherapeutic agent for hepatocellular carcinoma that is resistant to apoptosis and has elevated NCOA4 expression through ferritinophagy (Su and Lin, 2020).

SSPHI is a steroidal saponin derived from the Schizocapsa plantaginea Hance plant and is used as an anti-HCC agent. SSPH I demonstrated notable antiproliferative and antimigratory effects on HepG2 cells. The impact of SSPHI was partially reduced by the ferroptosis inhibitor ferrostatin-1 or the iron chelator ciclopirox. SSPH also triggered programmed cell death and halted the cell cycle at the G2/M phase (Zhou et al., 2020). Enhanced ROS generation, glutathione depletion, and malondialdehyde accumulation have been reported following SSPH I treatment, resulting in lipid peroxidation. Ferrostatin-1 and ciclopirox exhibited notable inhibitory effects against SSPH-I-induced lipid peroxidation. SSPHI increased the expression of SLC7A5, a negative regulator of ferroptosis. SSPHI increased the levels of TFR and Fpn proteins, resulting in the accumulation of Fe2+. Ferrostatin-1 and ciclopirox exhibited comparable antagonistic effects on SSPH I. Our research concludes that SSPHI triggers ferroptosis in HepG2 cells. Hence, SSPHI triggers ferroptosis in HepG2 cells by inducing iron overload (Huang et al., 2023).

Saikosaponin A (SA), a bioactive triterpenoid saponin isolated from Radix Bupleuri, exhibits strong anticancer effects in different tumor types. RNA sequence analysis revealed that SA mostly affected the glutathione metabolic pathway and suppressed the expression of cystine transporter solute carrier family seven member 11 (SLC7A11). SA elevated intracellular malondialdehyde and iron levels and reduced glutathione levels in HCC. Deferoxamine, ferrostatin-1, and GSH were effective in preventing SsA-induced cell death, whereas Z-VAD-FMK did not suppress SA-induced cell death in HCC. SA stimulates the production of activating transcription factor 3 (ATF3), which is essential for SA-induced ferroptosis and inhibition of SLC7A11 in hepatocellular carcinoma (Lan et al., 2023). Furthermore, SA caused an increase in ATF3 expression through the activation of endoplasmic reticulum stress, which indicated that ATF3-dependent cell ferroptosis is responsible for the anticancer effects of SA, suggesting that SA could be investigated as a ferroptosis inducer in hepatocellular carcinoma.

## 4 Nanoformulations for accelerated ferroptosis in liver cancer

To date, several tiny compounds that trigger ferroptosis have been reported. Nevertheless, these chemicals are hindered by significant disadvantages, such as low solubility, systemic toxicity, and limited tumor-targeting capability, which restrict their clinical efficacy. The concept that nanoparticles can induce ferroptosis and exhibit superior preclinical characteristics compared to small compounds, while also overcoming resistance to apoptosis, has introduced a new perspective for cancer therapy. Nanoparticles possess unique chemical and physical characteristics that allow them to be filled with anticancer medications or adorned with chemicals that specifically target tumors. These characteristics enable the use of medication combinations for treatment and specifically targeting tumors. This section provides a concise and detailed overview of the existing data on nanoparticles that can induce ferroptosis and possess unique characteristics that make them suitable for therapeutic applications, such as delivering anti-tumor drugs, targeting tumors, and modulating the immune system in liver cancer (Zaffaroni and Beretta, 2021) (Table 2).

**TABLE 2:** Nanomaterials targeting ferroptosis in liver cancer.

Nanomaterials	Mode of action	References
Sorafenib encapsulated in MIL-101(Fe) nanoparticles	Enhanced efficacy of ferroptosis-based therapies	Cao et al. (2020)
	Elevated lipid peroxidation and malondialdehyde levels	
	Reduced glutathione and glutathione peroxidase 4 levels	
Cu-hemin-PEG-lactose acid	Increased intracellular lipid reactive oxide species	Li et al. (2021b)
	Triggered ferroptosis	
	Enhanced BID (BH3 interacting domain death agonist), AIF (apoptosis-inducing factor, and EndoG (endonuclease G protein) expression	
MMSNs@Sorafenib	Reduced tumor cell growth	Tang et al. (2019)
	Enhanced intracellular lipid peroxide	
	Triggered ferroptosis	
AFN@Cell membrane	Increased intracellular lipid peroxide species	Liu et al. (2023)
	Enhanced ferroptosis and tumor suppression	
USFe^3+^ LA nanoparticles	Inhibited tumor spread	Zhao et al. (2024)
	Immunogenic cell death induction	
	Inhibition of tumor growth and distant metastasis	
	Better biocompatibility	
	Enhanced the immune response	
RF@LA-Fe-MOF	Targeted and enhanced ferroptosis	Zhang et al. (2023b)
	Strong anti-proliferative and anti-metastatic effects	
	Increased lipid peroxide formation	
ADM/Fe_3_O_4_-MS	Enhanced T2-weighted MRI properties	Chen et al. (2022c)
	Increased antitumor effectiveness	
	Triggered ferroptosis	
LDL-DHA nanoparticles	Suppressed growth of HCC xenografts	Ou et al. (2017)
	Elevated tissue lipid hydroperoxide levels	
	Reduced GPX4 expression	
	Enhanced ferroptosis	
AA/ASP-AZO-Fc (AAAF)	Increased ferroptosis in solid tumors	Liu et al. (2022b)
	Reduced glutathione (GSH) levels	
	Boosted cell absorption	
	Enhanced growth-inhibiting effect	
(SeNP)-loaded β-glucan nanotubes (BFP-Se)	Improved bioavailability of SeNPs	Cai et al. (2022)
	GSH depletion	
	Induction of cellular redox imbalance	
Fe3O4-PEI@HA-RSL3	Triggered ferroptosis	Liang et al. (2023b)
	Suppressed hepatoma cell growth	
	Boosted ROS generation	
AD-doped TME-responsive vesicles	Increased intracellular Fe^2+^ and •OH levels	Su et al. (2023)
	Intensified oxidative stress	
	Increased ferroptosis	
	Disruption of the antioxidant system	
	Inhibition of glutathione formation	
Hollow mesoporous Prussian blue nanoparticles	Cell death induction	Yue et al. (2022)
	Reduced cell count for chemotherapy	
	Enhanced reactive oxygen species generation	
GO-PEI-PEG/PD-L1 siRNA	Reduction of HCC tumors	Li et al. (2022b)
	Increased buildup of reactive oxygen species Increased ferroptosis	
	Stimulated adaptive immunity and tumor ferroptosis	
ExoSP94-lamp2b-RRM-multi-siRNA	Improved HCC responsiveness to sorafenib	Li et al. (2022c)
	Boosted sorafenib-induced ferroptosis	
	Enhanced deliverer of multiple siRNAs to HCC tissues	
Exosome-mimicking M1 nanovesicles	Depleted glutathione levels	Meng et al. (2023)
	Reducing the lipid antioxidant glutathione peroxidase-4	
	Induced ferroptosis in tumor cells	

A formulation of sorafenib encapsulated in MIL-101(Fe) nanoparticles was developed to enhance the efficacy of ferroptosis-based therapies for hepatocellular cancer. MIL-101(Fe) nanoparticles exhibit advantageous features such as drug loading, controlled release, peroxidase activity, biocompatibility, and T2 magnetic resonance imaging capability (Cao et al., 2020). The effectiveness of ferroptosis-based therapy for HCC was assessed through co-administration of the iRGD peptide in both laboratory tests and live subjects. Sorafenib-containing MIL-101(Fe) nanoparticles effectively triggered ferroptosis in HepG2 cells by elevating lipid peroxidation and malondialdehyde levels while decreasing glutathione and glutathione peroxidase four levels. *In vivo*, findings showed that sorafenib-loaded MIL-101(Fe) nanoparticles effectively suppressed tumor growth and reduced GPX-4 expression levels with minimal long-term harm (Liu et al., 2021). Concurrently, combining sorafenib-loaded MIL-101(Fe) nanoparticles with iRGD significantly hastened ferroptosis.

Sorafenib is an oral medication used as the initial treatment for hepatocellular carcinoma. However, the therapeutic efficacy of sorafenib is limited. An oral delivery system was created to enhance sorafenib absorption by hepatocellular carcinoma and trigger strong ferroptosis (Li Z. et al., 2021a). The platform consists of butyrate-modified nanoparticles, each containing sorafenib and salinomycin. Butyrate, a versatile ligand, interacts with monocarboxylate transporter 1 (MCT-1) to enhance transcytosis. MCT-1 is expressed differently on the apical and basolateral sides of the gut. It is substantially expressed on the surface of hepatocellular carcinoma cells but is expressed at low levels in normal hepatocytes. When taken orally, this platform enhances transepithelial transport in the intestine, drug accumulation in the liver, and uptake by HCC cells consistently and efficiently. Sorafenib depletes glutathione peroxidase four and glutathione in cancer cells, leading to the initiation of ferroptosis. Salinomycin increases intracellular iron levels and lipid peroxidation, leading to accelerated ferroptosis (Yu et al., 2023). This combination approach triggered significant ferroptosis damage and initiated a strong systemic immune reaction, resulting in the successful eradication of tumors and the development of systemic immunological memory. This study demonstrates a proof-of-concept for using an oral delivery method of ferroptosis inducers to potentially benefit hepatocellular carcinoma treatment.

Liver tumors are challenging to treat owing to their high aggressiveness and rapid development. Ferroptosis is anticipated to overcome the therapeutic challenges associated with liver tumors. The proposed technique aims to trigger ferroptosis in HepG2 cells using acid-degradable tumor-targeted nanosheets called Cu-hemin-PEG-lactose acid (CPLA). Once taken up by HepG2 cells, the CPLA nanosheets are broken down by mild acid, releasing Cu(II) and hemin (Yu et al., 2023). This process depletes intracellular GSH and increases the production of heme oxygenase 1 (HMOX1). The production of glutathione peroxidase 4 (GPX4) is decreased by reducing intracellular GSH levels by converting GSH into oxidized glutathione (GSSG) and excessive intracellular Fe^2+^ concentration via a substantial increase in HMOX1 expression. The combined effect of classical and non-classical pathways led to an increase in intracellular lipid reactive oxide species, triggering ferroptosis and enhancing the expression of BH3 interacting domain death agonist (BID), apoptosis-inducing factor (AIF), and endonuclease G proteins (EndoG). This synergistic technique shows a strong ability to induce ferroptosis and anticancer effects in living organisms, indicating promising possibilities for the therapeutic application of ferroptosis (Li K. et al., 2021b).

A recent study presented a novel one-pot procedure for producing manganese-silica nanoparticles (MMSNs) that can trigger ferroptosis in tumor cells by depleting intracellular GSH owing to the breakdown of MMSNs. Increasing the quantity of MnCl2 injected during the preparation resulted in a higher Mn doping level in the MMSNs. High concentrations of glutathione can cause the manganese-oxidation bonds in MMSNs to break, leading to rapid depletion of glutathione in the surrounding environment (Tang et al., 2019). Sorafenib was encapsulated into MMSNs to create MMSNs@SO, which enabled controlled drug release in the tumor microenvironment as a result of MMSNs degradation. A considerable decrease in tumor cell growth was achieved using MMSNs@SO, which consumed GSH and inhibited the formation of intracellular GSH in HepG2 cells. Reduced GSH levels caused glutathione peroxidase four to become inactive and led to an increase in intracellular lipid peroxide, perhaps triggering ferroptosis in hepatocellular carcinoma cells. Dual GSH-exhausting nanodrugs can cause ferroptosis in hepatocellular cancer cells (Tang et al., 2019).

There is a need for the strategic development of therapeutics based on ferroptosis involving the precise delivery of ferroptosis-inducing agents for hepatocellular carcinoma. Arsenic trioxide (ATO) effectively triggers ferroptosis in HCC cells, and this effect can be counteracted by the iron chelator deferoxamine, as demonstrated in our study. A drug delivery system, known as AFN@CM, was created by disguising ATO-loaded magnetic nanoparticles (Fe_3_O_4_) with HCC cell membranes to improve the effectiveness of ATO (Liu et al., 2023). Following AFN@CM therapy, glutathione peroxidase four was markedly suppressed, leading to a notable increase in intracellular lipid peroxide species in HCC cells. Enhanced ferroptosis and tumor suppression are evident in both laboratory settings and living organisms. Moreover, AFN@CM exhibited improved accumulation at tumor sites and achieved sustained tumor eradication in a mouse model owing to the cell membrane coating. AFN@CM showed effective therapeutic results in treating HCC and offers a reliable ferroptosis-based approach for safe HCC treatment (Liu et al., 2023).

The diversity of hepatocellular carcinoma and the intricate nature of the tumor microenvironment (TME) create obstacles to effective medication delivery and the success of combination or synergistic treatments (Sas et al., 2022). A metal-coordinated carrier-free nano-drug, USFe^3+^ LA NPs, was created for synergistic anti-HCC effects through ferroptosis. Ursolic acid (UA), a natural substance, was used to increase the susceptibility of tumor cells to sorafenib. The incorporation of cell penetration peptides and epithelial cell adhesion molecule aptamers on the surface-enhanced the uptake of USFe^3+^ LA NPs by HepG2 cells. Fe^3+^ ions can interact with intracellular hydrogen peroxide to produce harmful hydroxyl radicals (•OH) for chemodynamic therapy (CDT) and enhance ferroptosis through the cystine/glutamate antiporter system (System Xc−), leading to increased glutathione (GSH) depletion and reduced expression of glutathione peroxidase 4 (GPX4) (Zhao et al., 2024). Finally, the nanodrugs showed good biocompatibility and enhanced the immune response against PD-L1 by boosting the infiltration of cytotoxic T-cells. *In vivo* investigations showed notable inhibition of tumor growth and distant metastasis. This study presents a new approach using a metal-coordinated co-assembled carrier-free nano-delivery system for HCC combined therapy, focusing on cancer metastasis prevention and immunotherapy.

Inducing ferroptosis in cancer cells is a beneficial approach for inhibiting tumor proliferation and spread. The intrinsic ferroptosis suppressors GPX4 and FSP1 impair the effectiveness of ferroptosis therapies. A drug delivery system was created by incorporating Fe(III) into a metal-organic framework (MOF), loaded with small molecules RSL3 and iFSP1, and modifying it with the liver-targeting ligand lactobionic acid. Glutathione reduces Fe(III) to Fe(II) in hepatocellular carcinoma, triggering ferroptosis and breaking down RF@LA-Fe-MOF to release RSL3 and iFSP1, which act as inhibitors of GPX4 and FSP1, respectively. Simultaneous blocking of two ferroptosis suppressors at the same time significantly increases ferroptosis in hepatocellular carcinoma (Zhang et al., 2023b). This study successfully targeted and enhanced ferroptosis in HCC by utilizing a drug delivery system with two small-molecule ferroptosis enhancers.

Transarterial chemoembolization (TACE) is a primary palliative treatment for advanced hepatocellular carcinoma and a promising approach for cancer therapy (Zhang et al., 2023b). Drug-loaded microspheres (DLMs) used in clinical chemoembolization have issues with inconsistent particle sizes and unreliable treatment effectiveness. Homogeneous gelatin microspheres containing both adriamycin and Fe_3_O_4_ nanoparticles (ADM/Fe_3_O_4_-MS) were created using a high-voltage electrospray technique. This study validated the role of ferroptosis in the antitumor mechanism of ADM/Fe_3_O_4_-MS combined with microwave irradiation. The expression of the ferroptosis marker GPX4 decreased significantly, whereas that of ACSL4 increased significantly (Chen et al., 2022c). These findings confirmed that microwave-induced hyperthermia can enhance the effectiveness of ADM/Fe_3_O_4_-MS in fighting tumors by triggering ferroptosis. In addition, Fe_3_O_4_ nanoparticles can notably enhance TACE in HCC. This study verified the practicality of utilizing uniformly sized gelatin microspheres containing Fe_3_O_4_ nanoparticles and adriamycin to improve the effectiveness of TACE for HCC.

LDL nanoparticles containing docosahexaenoic acid have been found to specifically destroy liver cancer cells and inhibit the development of liver tumors in rats (Wen et al., 2016). Currently, there is limited knowledge regarding the cell death mechanisms through which LDL-DHA nanoparticles eliminate tumor cells. When treated with LDL-DHA, rat and human HCC cells underwent significant lipid peroxidation, glutathione depletion, and inactivation of the lipid antioxidant GPX4 before cell death. Treatment with inhibitors showed that HCC cells die regardless of apoptotic, necroptotic, or autophagic pathways but require the presence of cellular iron. Administering LDL-DHA directly into the tumor significantly suppressed the growth of HCC xenografts over an extended period. Hepatocellular tumors treated with LDL-DHA underwent necroptotic cell death, marked by elevated tissue lipid hydroperoxide levels and reduced GPX4 expression (Ou et al., 2017). An angelica polysaccharide-based nanocarrier material was synthesized to target hypoxic tumor microenvironments and utilize the new programmed cell death process of combined ferroptosis. The polymer micelles were hypothesized to function as natural liver-targeted drug delivery carriers because of the presence of ASP with liver targeting. The structure of AAAF was verified by 1H-NMR and FT-IR spectroscopy. The hydrophobic drug curcumin was encapsulated in polymer micelles named AAAF@Cur, which exhibited a uniform spherical shape. The *in vitro* release assay confirmed that AAAF@Cur could trigger the release of medication in response to hypoxia. Furthermore, a set of cell studies have verified that hypoxia may boost cell absorption and significantly enhance the growth-inhibiting effect on HepG2 cells (Liu et al., 2022b).

Increasing oxidative stress has emerged as a viable approach to successful cancer treatment. Drug resistance is encouraged, and potency is decreased by the overactive antioxidant systems found in tumor cells, which counteract this impact (Hayes et al., 2020). Another study presented a novel approach to cancer treatment by utilizing selenium nanoparticle (SeNP)-loaded β-glucan nanotubes (BFP-Se), which are naturally occurring triple-helix glucans generated from black fungus, to induce cellular redox imbalance and deplete glutathione (GSH). Metabolomics revealed that metabolic reactions related to BFP-Se are mostly linked to oxidative stress in hepatoma cells. BFP-Se was shown to deplete intracellular GSH, inhibit TXNIP/TRX and NRF2/GPX4-associated antioxidant system expression, and generate reactive oxygen species by interacting with intracellular H_2_O_2_ in both *in vivo* and *in vitro* experiments. This ultimately results in apoptosis and ferroptosis of hepatoma cells (Cai et al., 2022). Complex magnetic nanocube Fe_3_O_4_ modified with PEI and HA was successfully synthesized using the thermal decomposition method. RSL3 blocks the ferroptosis signal transduction pathway in cancer cells by inducing ferroptosis. Through HA-CD44 binding and an external magnetic field, the drug delivery system may be able to actively target tumor cells. Zeta potential investigation revealed that in the acidic environment of the tumor, Fe3O4-PEI@HA-RSL3 nanoparticles were more stable and evenly distributed. With increased treatment with Fe3O4-PEI@HA-RSL3 nanocubes, there was considerable suppression of the expression of genes linked to ferroptosis, including Lactoferrin, FACL 4, GPX 4, and ferritin (Liang et al., 2023b). Thus, the use of this ferroptosis nanomaterial for the treatment of hepatocellular carcinoma (HCC) offers enormous promise.

A nano platform has been developed to simultaneously deliver the anticancer medication sorafenib and the ferroptosis inducer hemin for combined ferroptosis-apoptosis therapy in advanced hepatocellular carcinoma. pH-sensitive vesicles (SH-AD-L) were created by combining amphiphilic dendrimers (AD) with liposomes to release sorafenib and hemin in the acidic tumor microenvironment (TME) in a regulated and pH-triggered manner because of the protonation of several amine groups in AD. SH-AD-L inhibits glutathione formation, leading to disruption of the antioxidant system (Su et al., 2023). It also increases intracellular Fe^2+^ and •OH levels, intensifying oxidative stress, all of which contribute to increased ferroptosis. This study showed that combining ferroptosis with apoptosis is effective in treating advanced HCC. Nanocarriers have demonstrated potential in medical delivery in recent years to address these issues efficiently. Sulfur oxide-loaded nanocarriers have been used to address the limitations of chemotherapy in the treatment of carcinoma. pH-sensitive hollow mesoporous Prussian blue nanoparticles (HMPB) were enclosed with SO, an inhibitor of multi-kinase and an accelerator of ferroptosis, to serve as carriers and enhance drug release. A pH-responsive chitosan (CS) layer was applied to the surface to prevent the medication from escaping and to enhance biocompatibility. Hence, HP/SO/CS nanocomposites were successfully designed to target tumor cells by boosting the permeability and retention (EPR) effect (Yue et al., 2022). Mice treated with nanocomposites in the *in vivo* experiments had the smallest tumor sizes and body weights and showed no apparent harm to normal tissues and organs. Collectively, these results suggested that nanocarriers effectively inhibited HCC cells.

Graphene oxide (GO)-PEI-PEG was synthesized to transport PD-L1 siRNAs to hepatocellular carcinoma (HCC) cells via the endocytosis-lysosome pathway. GO-PEI-PEG successfully targeted the mouse liver. In C57BL/6 mice, intrahepatic tumors were treated with GO-PEI-PEG/PD-L1 siRNAs via the tail vein. This treatment leads to the reduction of HCC tumors and enhances antitumor effectiveness when combined with oral sorafenib (Li et al., 2022b). It also increased the levels of Perforin, Gzmb, Ifng, Il-1b, and Tnfa in the tumors following the combination treatment. These therapies increase the buildup of reactive oxygen species and accelerate ferroptosis in HCC. These findings indicate that the combination of GO-PEI-PEG delivering PD-L1 siRNAs with oral sorafenib can stimulate adaptive immunity and tumor ferroptosis, offering a promising treatment for advanced HCC patients.

Sorafenib is a potent first-line medication authorized for treating advanced hepatocellular carcinoma. Exosomes were engineered to have a high capacity for carrying multiple siRNAs and specifically targeting hepatocellular carcinoma by combining the SP94 peptide and N-terminal RNA recognition motif (RRM) of U1-A with the exosomal membrane protein Lamp2b (Li et al., 2022c). The findings from both laboratory and animal trials suggest that HCC-targeted exosomes (ExoSP94-Lamp2b-RRM) can deliver multiple siRNAs to HCC tissues, boost sorafenib-induced ferroptosis by suppressing GPX4 and DHODH expression, and thereby improve HCC responsiveness to sorafenib, offering a new approach to address sorafenib resistance through the lens of ferroptosis. Silica nanoparticles (SiNPs) are widely produced and utilized artificial nanomaterials that have raised environmental health and safety (EHS) concerns owing to the growing risks posed by their exposure to the environment and humans. SiNPs induced ferroptosis in L-02 cells *in vitro*, as shown by decreased cell viability, disruption of mitochondrial membrane/cristae, increased phospholipid hydroperoxides (PL-OOH), accumulation of free ferrous iron in mitochondria and intracellularly, and impaired capacity for repairing lipid peroxidation (Liang et al., 2022). This research indicates that NCOA4-mediated ferritinophagy, a type of autophagy triggered by SiNPs, is a new mechanism for SiNP-induced hepatocyte ferroptosis and liver damage, reported for the first time.

Ferroptosis is a novel approach to combat the resistance to conventional cancer treatments by inducing deadly lipid peroxidation, which results in immunogenic cell death. The limited effectiveness of ferroptosis inducers and their low immunogenicity hinder their broader clinical use. Exosome-mimicking M1 nanovesicles (MNV) were created by repeatedly extruding M1 macrophages. In a mouse model of hepatocellular carcinoma, MNV@DHA effectively targeted tumor tissues, increased the presence of M1 macrophages and CD8^+^ T cells, and reduced the invasion of M2 macrophages. MNV@DHA, characterized by a positive feedback control between ferroptosis and immunological activation, demonstrated the most potent therapeutic effect *in vivo* (Meng et al., 2023). The combination of ferroptosis and immunomodulation using polyunsaturated fatty acids from the diet and manufactured exosome-mimicking nanovesicles shows potential as a promising method to enhance pharmaceutical strategies for cancer treatment.

**FIGURE 2:**
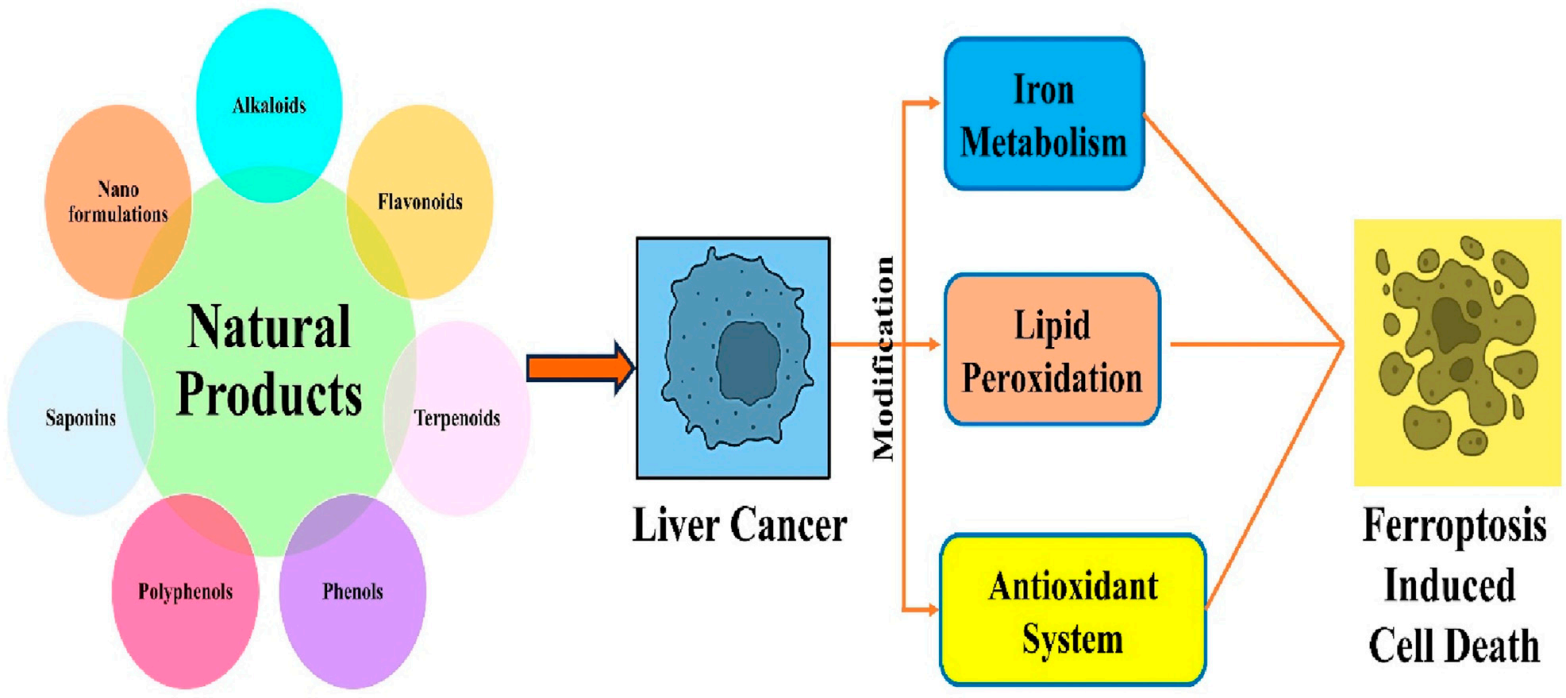
Ferroptosis in liver cancer mediated by plant-based compounds.

This review offers comprehensive insights into using natural chemicals to target ferroptosis in hepatocellular carcinoma (Figure 2). This will assist future researchers in understanding powerful therapeutic strategies for managing liver cancer. This review has identified natural chemicals that specifically target ferroptosis in liver cancer and provides a summary of nanoformulations that improve the absorption and delivery of medications for more effective treatment of liver cancer.

## 5 Conclusion

Ferroptosis is increasingly being recognized as a significant factor in the initiation and progression of cervical cancer. Targeting it is a promising cancer prevention and treatment method in clinical settings. A thorough reassessment and update of the molecular pathways involved in targeting ferroptosis in cancer using natural products is required, owing to recent advancements in research. We conducted a thorough search and analysis of pertinent literature using the Web of Science database, with a specific emphasis on the regulatory effects of natural products and their active components on the management or prevention of cervical cancer through the modulation of ferroptosis. Many natural products and their active components have been found to induce cervical cancer cell death by modulating the System Xc^−^-GPX4 axis and lipid, mitochondrial, and iron metabolism in a process known as ferroptosis. Natural materials can enhance the effectiveness of chemotherapy by inducing ferroptosis in cervical cancer cells owing to their polypharmacological properties. The molecular processes regulating ferroptosis by natural products will facilitate the development of natural anticancer cancer therapies by targeting ferroptosis. This review will be highly useful in cancer therapeutics by utilizing detailed insights into the use of natural compounds for developing potent lead candidates for the efficient management of liver cancer.

## Figures and Tables

**Table:** 

**GSH**	glutathione
**RCD**	regulated cell death
**ROS**	reactive oxygen species
**GPX4**	glutathione peroxidase 4
**PTGS2**	prostaglandin synthase 2
**NADPH**	nicotinamide adenine dinucleotide phosphate
**NRF2**	nuclear factor erythroid 2-related factor 2
**TF**	transcription factor
**MT-1G**	Metallothionein-1G
**TACE**	Transcatheter arterial chemoembolization
**HCC**	hepatocellular carcinoma
**AFP**	alpha-fetoprotein
**Rb**	retinoblastoma
**r1R**	sigma-1 receptor
**HIC1**	HIC ZBTB Transcriptional Repressor 1
**HNF4A**	HNF4A hepatocyte nuclear factor 4 alpha
**NQO1**	NAD(P)H Quinone Dehydrogenase 1
**SLC1A5**	Solute Carrier Family 1 Member 5
**ACACA**	Acetyl-CoA Carboxylase Alpha
**ACSL3**	Acyl-CoA Synthetase Lon Chain Family Member 3
**CARS1**	Cysteinyl-TRNA Synthetase 1
**CISD1**	CISD1 - CDGSH iron-sulfur domain-containing protein 1
**G6PD**	Glucose-6-Phosphate Dehydrogenase
**SLC1A5**	Solute Carrier Family 1 Member 5
**RRM2**	ribonucleotide Reductase Regulatory TP53 Inducible Subunit M2B
**PVT1**	plasmacytoma variant translocation 1
**HERPUD1**	(Homocysteine inducible ER protein with ubiquitin like domain 1
**ATF3**	Activating transcription factor 3
**SAG**	Sanguinarine
**ULK1**	Unc-51-like autophagy-activating kinases 1
**LC3B**	Tubule-associated protein 1 light chain 3 beta
**ATG4**	Autophagy-regulating protease 4
**mTOR**	mammalian target of rapamycin
**TBK1**	TANK-binding kinase 1
**TCM**	Traditional Chinese Medicine
**PTL**	Parthenolide
**NAC**	N-acetylcysteine
**SA**	Saikosaponin A
**SLC7A11**	solute carrier family 7 member 11
**MCT-1**	monocarboxylate transporter 1
**CPLA**	Cu-hemin-PEG-lactose acid
**ATO**	Arsenic trioxide
**TME**	tumor microenvironment
**UA**	Ursolic acid
